# Image Characteristics of Virtual Non-Contrast Series Derived from Photon-Counting Detector Coronary CT Angiography—Prerequisites for and Feasibility of Calcium Quantification

**DOI:** 10.3390/diagnostics13223402

**Published:** 2023-11-08

**Authors:** Franziska M. Braun, Franka Risch, Josua A. Decker, Piotr Woźnicki, Stefanie Bette, Judith Becker, Katharina Rippel, Christian Scheurig-Münkler, Thomas J. Kröncke, Florian Schwarz

**Affiliations:** 1 Clinic for Diagnostic and Interventional Radiology and Neuroradiology, University Hospital Augsburg, Stenglinstr. 2, 86156 Augsburg, Germany; franziska.braun@uk-augsburg.de (F.M.B.); josua.decker@uk-augsburg.de (J.A.D.); pjotr.a.woznicki@gmail.com (P.W.); stefanie.bette@uk-augsburg.de (S.B.); judith.becker@uk-augsburg.de (J.B.); christian.scheurig@uk-augsburg.de (C.S.-M.); florian.schwarz@gmail.com (F.S.); 2 Department of Diagnostic and Interventional Radiology, University Hospital Würzburg, Oberdürrbacher Straße 6, 97080 Würzburg, Germany; 3 DONAUISAR Clinic Deggendorf, Perlasberger Str. 41, 94469 Deggendorf, Germany; 4 Medical Faculty, Ludwig Maximilian University of Munich, Geschwister-Scholl-Platz 1, 80539 Munich, Germany

**Keywords:** photon-counting detector CT, virtual non-contrast imaging, coronary artery calcium quantification, cardiac imaging

## Abstract

In photon-counting detector CT (PCD-CT), coronary artery calcium scoring (CACS) can be performed using virtual non-contrast (VNC) series derived from coronary CT angiography (CCTA) datasets. Our study analyzed image characteristics of VNC series in terms of the efficacy of virtual iodine “removal” and image noise to determine whether the prerequisites for calcium quantification were satisfied. We analyzed 38 patients who had undergone non-enhanced CT followed by CCTA on a PCD-CT. VNC reconstructions were performed at different settings and algorithms (conventional VNC_Conv_; PureCalcium VNC_PC_). Virtual iodine “removal” was investigated by comparing histograms of heart volumes. Noise was assessed within the left ventricular cavity. Calcium was quantified on the true non-contrast (TNC) and all VNC series. The histograms were comparable for TNC and all VNC. Image noise between TNC and all VNC differed slightly but significantly. VNC_Conv_ CACS showed a significant underestimation regardless of the reconstruction setting, while VNC_PC_ CACS were comparable to TNC. Correlations between TNC and VNC were excellent, with a higher predictive accuracy for VNC_PC_. In conclusion, the iodine contrast can be effectively subtracted from CCTA datasets. The remaining VNC series satisfy the requirements for CACS, yielding results with excellent correlation compared to TNC-based CACS and high predicting accuracy.

## 1. Introduction

Electrocardiogram-synchronized, non-enhanced computed tomography (NECT) scans of the heart are the primary non-invasive imaging modality for assessing the presence and extent of coronary artery calcification [1], a direct measure of an individual’s burden of coronary atherosclerosis [2]. The coronary artery calcium score (CACS) has substantial prognostic value for predicting major adverse cardiovascular events and even long-term mortality in both asymptomatic [3,4,5,6] and symptomatic individuals [7,8], and it enhances cardiovascular risk stratification beyond traditional risk factor models [9,10,11]. NECT for calcium scoring may be performed as a stand-alone examination in asymptomatic individuals [12]. However, in most cases, it is followed by coronary CT angiography (CCTA) to visualize the coronary artery lumen, including potential stenoses and non-calcified plaques [13]. Because of the introduction of dual-energy computed tomography, virtual non-contrast (VNC) series can be derived from contrast-enhanced CT datasets via material decomposition using iodine and water as reference materials [14]. Several studies have validated the feasibility and accuracy of calcium scores based on VNC series derived from CCTA scans acquired with dual-energy CT [15,16,17,18,19,20]. Photon-counting detector CT (PCD-CT) is a novel spectral CT technology. Over the energy-integrating detector CT, PCD-CT offers higher spatial resolution, the elimination of electronic noise, and increased contrast-to-noise ratio [21]. Importantly, PCD-CT data exhibit intrinsic spectral information. Several recent studies have demonstrated that spectral PCD-CT information can be harnessed to estimate calcium quantities on CCTA datasets [22,23] or late enhancement cardiac scans [24]. Emrich et al. demonstrated a high correlation in CACS between VNC and the reference standard and an improved agreement for VNC_PC_-derived scores [22]. In addition, Fink et al. found in their phantom study that CACS accuracy on VNC_PC_ is influenced by the level of iteration and virtual monoenergetic keV during reconstruction [23]. Importantly, neither study performs image characteristic analysis of the intermediary non-iodine attenuation component dataset, which would be crucial for the validity of the argument proposed in either study. The aim of this study was therefore to close this gap by systematically investigating the image characteristics of the intermediary VNC series. To this end, the efficacy of iodine “removal” and noise properties, as well as measures of calcium (score and volume) of various VNC series derived from the CCTA scan, were intraindividually compared with assimilable parameters of true non-contrast (TNC) series.

## 2. Materials and Methods

The protocol of this retrospective, single-center study was approved by our institutional review board (project nr. 21-0773, 10/2021), which waived the necessity to obtain study-specific informed consent.

### 2.1. Study Population

We screened all patients for study inclusion who had undergone our standard scan protocol prior to transcatheter aortic valve replacement in July and August 2021 on a first-generation PCD-CT scanner. The protocol includes an unenhanced scan of the heart followed by a CCTA. Exclusion criteria were defined as follows: (1) scan protocol deviation or incompleteness; (2) missing raw data (required for uniform image reconstruction); (3) errors during reconstruction; and (4) severe motion artifacts of the coronary arteries that preclude correct CACS. In patients with coronary artery stents, the stent segments and all coronary segments distal to the stent were excluded from further analysis.

### 2.2. Scan Protocol and Reconstruction Settings

All scans were performed on a first-generation dual-source PCD-CT system (NAEOTOM Alpha, Siemens Healthcare GmbH, Erlangen, Germany). As a component of our CT protocol, all patients underwent a NECT of the heart followed by a contrast-enhanced CT angiography (CTA) of the aorta and iliac arteries. The CTA scan range encompassed the internal carotid arteries to the distal common femoral artery. Scanning was electrocardiogram-triggered to ensure diastolic acquisition of the heart and coronary arteries. Both NECT and CTA examinations were performed at a tube voltage of 120 kVp, a gantry rotation time of 0.25 s, a high pitch of 3.2, and a collimation of 144 × 0.4 mm^2^. Patients did not receive betablockade or nitroglycerine prior to CT, in accordance with the recommendations for preprocedural CT prior to transcatheter aortic valve replacement [25]. For the CTA scan, a triphasic contrast material injection protocol was used as previously described [26], with 90 mL of contrast material in total (Ultravist 300, Bayer Vital GmbH, Leverkusen, Germany) and an injection rate of 5 mL/s, and a 50 mL saline chaser.

TNC from NECT and VNC_Conv_ series from CTA data were reconstructed at the scanner console, VNC_PC_ series were reconstructed on a dedicated workstation (ReconCT, Version 15.0, Siemens Healthcare GmbH, Erlangen, Germany). For TNC series, virtual monoenergetic images at 70 keV were generated using a regular quantitative kernel (Qr36), a slice thickness of 3.0 mm, an increment of 1.5 mm, and a field-of-view of 180 × 180 mm. From the CCTA dataset, several VNC series were reconstructed, again with a field-of-view of 180 × 180 mm, covering the heart. Reconstruction settings varied in kernel (Qr36 vs. Br36), strengths of iterative reconstruction (Q3 vs. Q4), and slice thickness/increment (0.4 mm/0.2 mm vs. 1.0 mm/0.4 mm). Two algorithms for virtual subtraction of contrast media were compared: the conventional VNC_Conv_ and the calcium-preserving VNC_PC_ (PureCalcium) algorithm. Detailed reconstruction parameters for each series can be taken from Table 1.

### 2.3. Image Analysis

A comprehensive analysis of the various VNC series and the TNC series was conducted and involved three key components: (1) assessment of the efficacy of virtual iodine “removal”; (2) image noise measurements; and (3) quantification of coronary artery calcium.

To assess the efficacy of virtual iodine “removal”, the image volumes of each patient were transformed to obtain isotropic 1 × 1 × 1 mm voxels and registered, and a semi-manual segmentation of the whole heart was carried out with the open-source software 3D Slicer (Version 4.11) (3D Slicer, www.slicer.org). Histograms of CT values and their proportions exceeding a threshold of 130 HU were compared between CTA, TNC, VNC_Conv_, and VNC_PC_. As the different reconstruction settings were not expected to influence the virtual iodine “removal”, VNC^1^ series were examined as representatives.

To measure image noise, three circular regions of interest (ROIs) with a diameter of 15 mm each were positioned in the left ventricular cavity on the CTA reconstruction of each patient, carefully avoiding papillary muscles, trabeculations, and the ventricular wall. These ROIs were then automatically copied to all VNC series and to the TNC series of the same patient. The standard deviation of CT values within these ROIs served as a measure for image noise.

Quantification of coronary artery calcium was performed by a board-certified radiologist. To determine inter-reader correlation, an independent reading was performed by a second radiologist, who evaluated 10 randomly selected patients. Calcifications were quantified using Agatston score and calcium volume on a per-patient and per-vessel level. A commercially available semi-manual calcium scoring software (Version VB60) (Syngo.CT CaScoring, Siemens Healthcare GmbH, Erlangen, Germany) was used for analysis, with a detection threshold of 130 HU. Both observers were blinded to the patients’ identity, all clinical data, and the reconstruction algorithms and series names. The time interval between the analyses of the TNC and VNC series was at least one week.

### 2.4. Radiation Metrics

For radiation dose estimation, the volumetric computed tomography dose indices (CTDI_vol_) and the dose length products of the NECT and CTA were retrieved from the automatically recorded dose report. Effective radiation doses of the NECT were estimated by multiplying the respective dose length product with a standard conversion coefficient for adult chest CT (0.017 mSv/mGy × cm). For the CTA, we used the mean of the standard conversion coefficients of the chest (0.017 mSv/mGy × cm), abdomen (0.015 mSv/mGy × cm), and pelvis (0.019 mSv/mGy × cm) for effective dose calculation (0.017 mSv/mGy × cm) [27].

### 2.5. Statistical Analysis

Python (version 3.9) was utilized for statistical analysis in this study. Binary data are represented as absolute frequencies and proportions. Continuous data were tested for normal distribution using the Shapiro–Wilk test. The paired *t*-test and the Wilcoxon signed rank test were used to test for differences, and Pearson and Spearman correlation were used to test for similarities for parametric and non-parametric data, respectively. Observer agreement was calculated via intraclass correlation coefficient for single fixed raters (ICC3). For all linear regression related presentations and calculations, data were square root transformed prior to analysis to approximate normal distribution and to improve homoscedasticity. The coefficient of determination (r^2^) was used to rate the linear regression. To determine the predictive accuracy of calcium quantities on VNC series, a 10,000-fold bootstrap with a linear regression model was conducted. The mean absolute error (MAE) was calculated as the absolute difference between the predicted, back-transformed TNC value and the original TNC calcium quantity. *p*-values < 0.05 were considered to indicate statistical significance.

## 3. Results

### 3.1. Patient Baseline Characteristics

A total of 50 patients were primarily enrolled, and 12 patients had to be excluded from analysis due to the predefined exclusion criteria (*n*(1) = 3; *n*(2) = 1; *n*(3) = 1; *n*(4) = 6). The final analysis included 38 patients (22 men (57.9%)) with a median age of 80.0 (75.3–82.8) years. Table 2 summarizes patient characteristics.

All patients underwent CT imaging due to aortic valve disease: 36 patients suffered from severe aortic stenosis (aortic valve area 0.7 ± 0.2 cm^2^); 1 patient suffered from severe combined aortic valve disease; and 1 patient was planned for combined mitral and aortic valve intervention. Of the 38 patients, 3 presented with severe stenosis of an implanted biological aortic valve.

There was no significant difference in heart rates between the NECT and CTA scans, which were 73 (62–83) bpm and 72 (61–80.0) bpm, respectively (*p* = 0.12).

**Figure 1:**
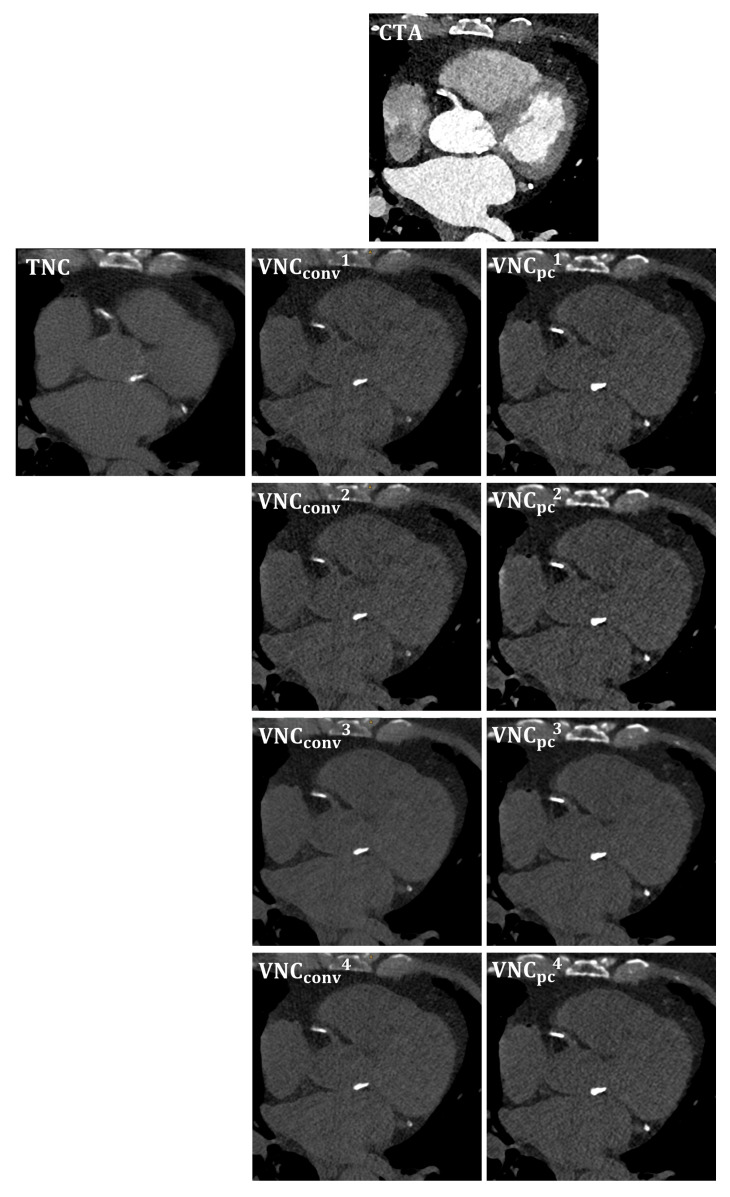
Corresponding image examples for TNC and all reconstructed VNC series, respectively. VNC_Conv_ = conventional virtual non-contrast; VNC_PC_ = pure calcium.

In the presence of coronary artery stents, the calcium scoring analysis excluded the coronary segment with the stent and all distal segments (left main coronary artery: n(LM) = 1; left anterior descending artery: *n*(LAD) = 5; circumflex artery: *n*(CX) = 1; right coronary artery: *n*(RCA) = 4). Three of the patients showed no measurable calcium volume in the coronary arteries. Based on the TNC series, the median Agatston and volume scores were 934 (167–1991) and 811 (200–1623) mm^3^ on a per per-patient level, respectively. Representative images are provided in Figure 1.

### 3.2. Iodine Removal

**Figure 2:**
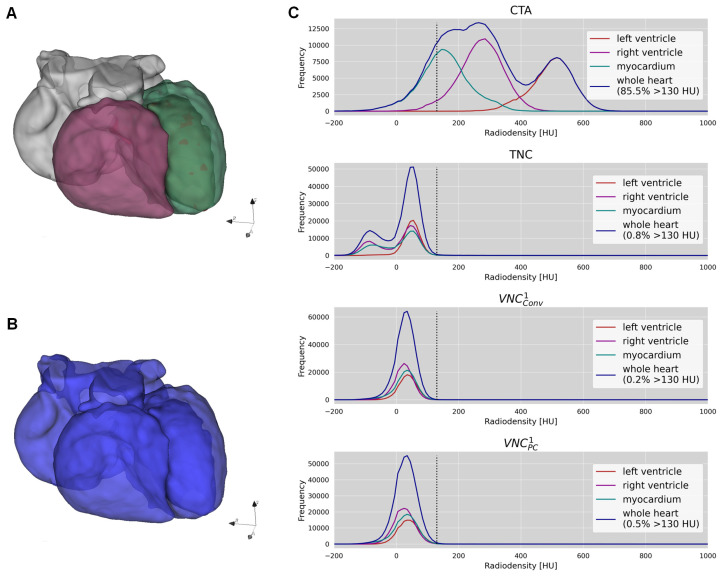
Effectiveness of iodine removal. (**A**) demonstrates the segmentation of the left ventricle, right ventricle, and myocardium. (**B**) shows the segmentation of the whole heart including the atria. (**C**) exhibits the histograms based on the heart segments for CTA, TNC, VNC_Conv_^1^, and VNC_PC_^1^. For the whole heart, the histogram proportion exceeding 130 HU (marked by the dotted line) is given.

As for evaluating the effectiveness of virtual iodine removal in VNC series, Figure 2 illustrates the method employed and the resulting histograms of the voxel CT-value distribution analysis. This figure highlights the presence of three distinct CT value peaks in the CTA heart histogram, with the highest CT values being observed within the left ventricle, followed by the right ventricle and the myocardium. In the TNC and VNC^1^ histograms, these peaks overlap, and barely any CT values exceed the threshold of 130 HU.

While the median proportion of CT values > 130 HU in whole-heart histograms was 82.2 (77.4–86.4%) for CTA, this proportion significantly decreased to 0.2 (0.1–0.6%) for VNC_Conv_^1^ and 0.7 (0.4–1.4%) for VNC_PC_^1^. With a median proportion of 0.6 (0.4–1.2%) for TNC, there was no significant difference to VNC_PC_^1^ (*p* = 0.4) but to VNC_Conv_^1^ (*p* < 0.01).

### 3.3. Image Noise

**Figure 3:**
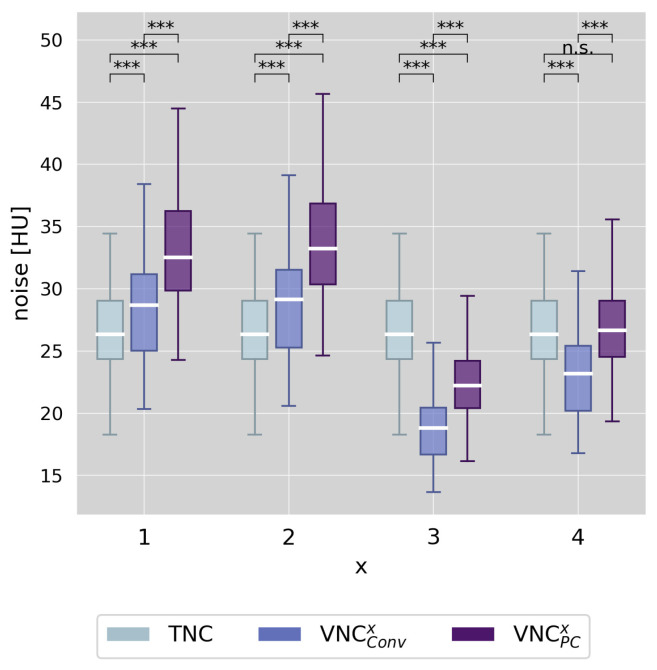
Boxplots of the noise levels measured as standard deviation of CT values in three circular regions of interest within the left ventricle comparing true non-contrast and virtual non-contrast (conventional and pure calcium) series, and differentiating between the different reconstruction settings of VNC^x^ (x = 1–4). Stars mark significant differences, as *** = *p* < 0.001, and n.s. marks no significant difference.

Regarding image noise, the results of the measurements are visualized in Figure 3. The measured noise level on TNC series was, on average, 26.4 ± 4.1 HU. Notably, image noise differed significantly between TNC and VNC (all *p*-values < 0.001), with the only exception being VNC_PC_^4^. The VNC reconstruction settings VNC^1^ and VNC^2^ resulted in rather higher noise levels (∆ < +3 HU for VNC_Conv_^1,2^ and ∆ < +7 HU for VNC_PC_^1,2^), and the VNC reconstruction settings VNC^3^ and VNC^4^ resulted in rather lower noise levels compared to TNC (∆ > −8 HU for VNC_Conv_^3,4^ and ∆ > −5 HU for VNC_PC_^3^).

### 3.4. Calcium Scoring

**Figure 4:**
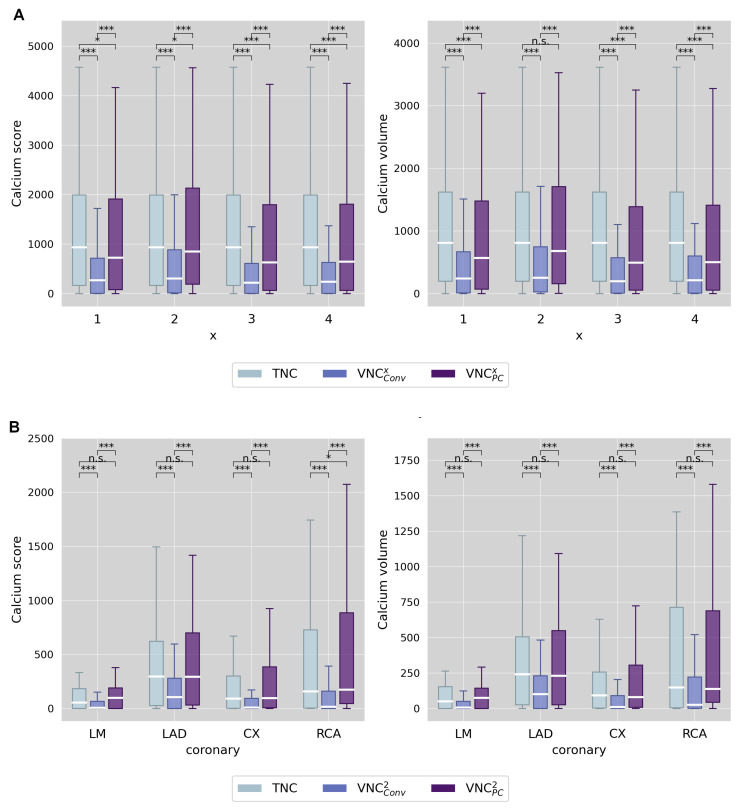
Boxplot of measured calcium quantities (score and volume) comparing TNC and VNC (conventional and pure calcium) series. (**A**) differentiates between the different reconstruction settings of VNC^x^ (x = 1–4) and (**B**) differentiates between the different coronary arteries considering only VNC^2^ series. Stars mark significant differences, as * = *p* < 0.05 and *** = *p* < 0.001, and n.s. marks no significant difference.

Observer agreement for calcium scores and calcium volumes was excellent for TNC and all VNC series, both on a per-patient and a per-vessel level (ICC3 agreement > 0.99). Analysis of the TNC series revealed three patients with a calcium score of zero. One of these patients was a false positive (TNC calcium score = 0; VNC calcium score > 0) in VNC_Conv_^2^, all three patients were false positives in VNC_PC_^2^, and two of them were false positives in VNC_PC_^1,3,4^. For VNC_Conv_ series, the discrepancies were small (Agatston score < 2), and for VNC_PC_ series, moderate (Agatston score up to 90). False negative results (TNC calcium score > 0; VNC calcium score = 0) occurred in a total in four patients: four times for VNC_Conv_^3^, three times for VNC_Conv_^4^, and once for VNC_PC_^3^. However, the respective TNC-based Agatston scores were small in all these cases (once < 10; twice < 80; and once < 160). The boxplot in Figure 4A shows the results on a per-patient level.

The total Agatston scores and calcium volume in the TNC datasets were 934.4 (166.8–1990.6) and 811.4 (199.6–1623.4), respectively. VNC_Conv_ series significantly underestimated calcium quantities (all *p*-values < 0.001). VNC_Conv_^2^ showed the smallest absolute difference to TNC, with a median score of 637 and a volume of 562 mm^3^.

The VNC_PC_ series also differed significantly from TNC, albeit to a much smaller extent. Again, VNC_PC_^2^ achieved the best results with the smallest absolute difference of 82 (score) and 80 mm^3^ (volume). Figure 4B presents the respective results on the per-vessel level for VNC_Conv_^2^ and VNC_PC_^2^.

**Figure 5:**
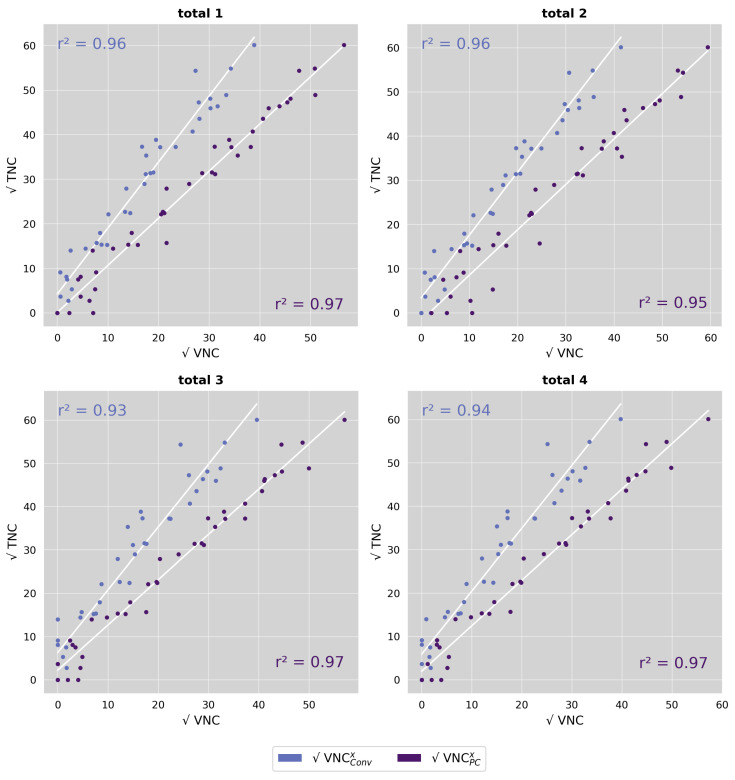
Linear regression analyses of TNC and VNC (conventional and pure calcium) series shown for the Agatston score on a per-patient level for the different reconstruction settings of VNC^x^ (x = 1–4). r^2^ = coefficient of determination.

**Figure 6:**
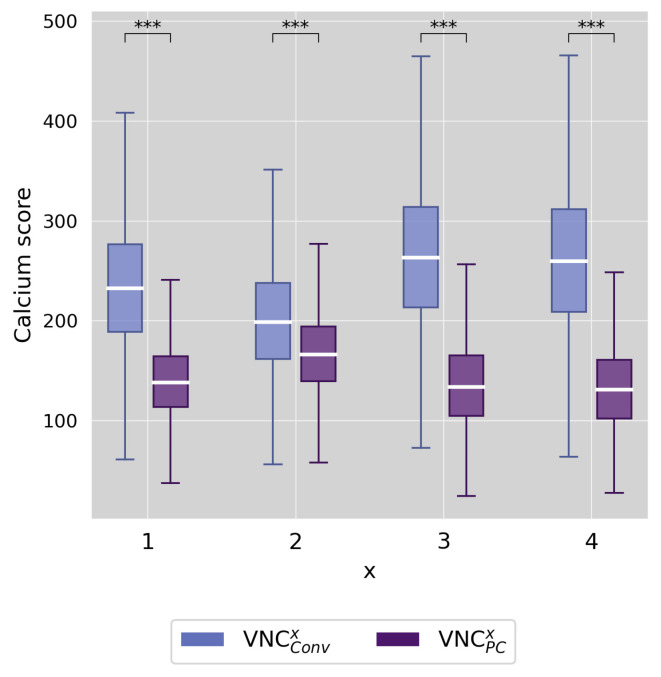
Boxplots of the mean absolute error between the predicted Agatston scores based on VNC (conventional and pure calcium) and Agatston scores derived from TNC series on a per-patient level resulting from the 10,000-fold bootstrapping analysis for the different reconstruction settings of VNC^x^ (x = 1–4). Stars mark significant differences with *** = *p* < 0.001.

Despite the differing absolute calcium quantity values, linear regression analysis showed excellent correlations between TNC and VNC, both globally (r^2^ > 0.93), as demonstrated in Figure 5, and on the per-vessel level (r^2^ > 0.85), regardless of reconstruction algorithm, reconstruction setting, or calcium quantity. However, Figure 6 shows that the mean absolute error between the predicted calcium quantity based on VNC measurements, and the true calcium quantity based on TNC measurements was significantly smaller for VNC_PC_ compared to VNC_Conv_ for all reconstruction settings (all *p*-values < 0.001). Among VNC_Conv_, VNC_Conv_^2^ achieved the highest predictive accuracy with a median absolute error of 199 (162–238) in Agatston and 152 (127–179) mm^3^ in volume score. All VNC_PC_ reconstructions except VNC_PC_^2^ showed similar low median absolute errors of <138 in Agatston and <110 mm^3^ in volume score.

### 3.5. Radiation Dose

For the NECT and CTA scans, dose length products were 31.8 (24.0–38.7) mGy × cm and 330.0 (256.5–412.3) mGy × cm, with corresponding effective doses of 22.3 ± 6.8 mSv and 64.5 ± 18.2 mSv and CTDI_vol_ of 1.5 (1.3–1.9) mGy and 4.4 (3.6–5.2) mGy, respectively.

## 4. Discussion

Our study systematically investigated the potential of spectral data acquired during coronary CT angiography on a photon-counting detector CT for distinguishing iodine and non-iodine attenuation components and quantifying calcium in CT angiography datasets. Our main findings are as follows: (1) virtual non-contrast series derived from CT angiography datasets exhibit a highly effective subtraction of the iodine attenuation component (i.e., contrast material): (2) the resulting virtual non-contrast series have suitable noise properties for a HU-threshold-based calcium quantification; (3) the absolute calcium quantities derived from virtual non-contrast series differ significantly from the absolute values measured on true non-contrast scans but show excellent correlation with the reference standard; and (4) the calcium-preserving virtual non-contrast algorithm VNC_PC_ outperforms the conventional algorithm VNC_Conv_ by achieving comparable absolute scores to the ground truth on TNC and yielding a higher predictive accuracy.

Due to their distinct mechanistic properties, PCD-CT systems generate spectral information about the tissue examined. Similar to earlier work on dual-energy CT, this can be utilized to derive VNC series from contrast-enhanced scans, such as CTA studies, via material decomposition. In VNC series, CT values represent the non-iodine attenuation component of each voxel, with values > 130 HU primarily attributable to calcium. This permits calcium quantification, akin to earlier work on dual energy CT [15,16,17,18,19,20,28]. Employing such a technique in the workup of coronary artery disease would have the advantage of eliminating the need for a dedicated non-enhanced acquisition, thereby reducing procedure time and overall radiation dose.

Presently, there remains a scarcity of literature regarding the implementation of this method on spectral PCD-CT data. Emrich et al. [22] and Fink et al. [23] have demonstrated a strong correlation between calcium quantities obtained from CCTA-derived VNC series and actual calcium quantities obtained from TNC series. In addition, utilizing a novel algorithm that produces virtual non-contrast pure-calcium (VNC_PC_) series by selectively subtracting the iodine attenuation component, calcium quantities measured on CTA datasets exhibited a high degree of concordance with actual calcium quantities. Despite the remarkable findings of these studies, neither one convincingly addressed the image features of VNC series, which must satisfy specific requirements for the validity of the aforementioned correlation or agreement to be unambiguously demonstrated. Our study closes this gap in knowledge.

VNC series, which are suitable for calcium quantification and interchangeable with TNC series, must satisfy at least three requirements: (1) effective “virtual removal” of the iodine attenuation component to generate a VNC dataset; (2) noise properties within the VNC dataset that permit HU-threshold-based calcium quantification, which necessitates low standard deviations in CT values in normal soft tissue; and (3) preservation of calcium. Failure to fulfill requirements (1) and (2) could result in false positives or inappropriately high calcium scores, while failure to meet requirement (3) could lead to false negatives or inaccurately low calcium scores.

To assess the fulfillment of requirement (1), we performed an extensive three-dimensional comparative analysis of the CT-value distribution for the entire heart in all relevant series (CTA, TNC, VNC_Conv_^1^, and VNC_PC_^1^ series). As anticipated, the CT-value histograms of the CTA datasets displayed mostly trimodal distribution (left ventricle, right ventricle, myocardium). Conversely, VNC series exhibited unimodal distributions closely resembling TNC series. These stark similarities in CT value distribution, both qualitatively and quantitatively, between TNC and VNC series strongly indicate a highly efficacious removal of the iodine attenuation component.

To assess the fulfillment of requirement (2), we evaluated the distribution of CT values within ROIs located in the left ventricle on the TNC and all VNC series. Calcium scoring is traditionally performed on 3-mm slices as this provides an optimal balance between image noise and calcium sensitivity [29]. Our reference standard TNC was acquired accordingly. Since VNC series are derived from underlying CCTA datasets at a significantly higher CTDI_vol_, we expected image noise to be comparable at much lower slice thicknesses; thus, we employed either 0.4 mm or 1.0 mm. Using additional variations in reconstruction kernels and strengths of iterative reconstructions, we derived four distinct series from the CCTA dataset for each VNC algorithm. Our analysis of image noise revealed significant differences between TNC and all VNC series. Notably, despite the low slice thickness of 0.4 mm, VNC^1,2^ series demonstrated only marginally higher image noise than TNC series. In VNC^3,4^ series (1.0-mm slice thickness), noise was even lower than in TNC series. In summary, these findings highlight that with the appropriate selection of VNC settings, requirement (2) can be easily met.

To evaluate the fulfilment of requirement (3), we used an indirect method of proof by comparing calcium quantities obtained from VNC series with those derived from TNC series, the reference standard. Our results, consistent with prior research on dual-energy CT, demonstrate that calcium quantities on VNC_Conv_ series consistently underestimate references values, whereas VNC_PC_ results in comparable absolute values. Notably, only a few cases of false negatives or false positives were found. We observed that VNC_Conv_ rather produces false negatives, while VNC_PC_ rather produces false positives, which can be explained by the different VNC reconstruction algorithms. However, both algorithms exhibit excellent correlation for both the entire coronary tree, as well as for individual coronary arteries and for calcium scores and volumes. Nonetheless, the 10,000-fold bootstrap cross-validation shows a higher predictive accuracy of VNC_PC_ for actual TNC calcium quantities.

Summarizing our results on the VNC dataset characteristics, the most favorable approach was the use of 0.4-mm reconstructions in combination with a high level of iterative reconstruction (QIR 4). Surprisingly, a regular-body Kernel (Br36f) yielded slightly superior results to the dedicated quantitative Kernel (Qr36).

In essence, our findings corroborate the conclusions drawn by Emrich et al. regarding the remarkable correlation and strong predictive value of calcium quantities measured on VNC series with actual calcium quantities. However, we go a step further in filling the remaining gap in knowledge by demonstrating that VNC series employed for this purpose are nearly indistinguishable from NECT regarding the presence of contrast material and exhibit optimal noise characteristics for HU-based calcium quantification. It is only when these requirements are met that the correlation of calcium quantities attains the logical validity as suggested.

Some limitations of our study merit consideration. First, with 38 patients, our study cohort was relatively small, and further studies with a larger study group should follow to confirm our results. Another limitation might be that our study cohort was examined using a high-pitch acquisition mode irrespective of the individual heart rate. Patients did not receive betablockade or nitroglycerine. Theoretically, one could expect higher proportions of CT scans affected by motion artifacts. To address this potential objection, patients with marked motion artifacts of the coronary arteries were excluded from further analysis.

## 5. Conclusions

In conclusion, our results show that the iodine-based attenuation component can be effectively subtracted from photon-counting detector coronary CT angiography datasets, and that the remaining non-iodine attenuation component satisfies all the mentioned requirements for calcium quantification, yielding coronary artery calcium quantities with excellent correlation to the reference standard TNC. For the conventional VNC_Conv_ algorithm, the best results were obtained by the use of ultra-thin-slice reconstructions (0.4 mm) in combination with a high level of iteration (QIR4). The calcium-preserving VNC_PC_ algorithm was not influenced by the reconstruction settings tested in this study, and it even outperformed VNC_Conv_. Therefore, calcium scoring on VNC_PC_ raises the prospect of substituting true non-contrast scans with a virtual non-contrast reconstruction to save radiation dose, time, and cost.

## Figures and Tables

**Table 1:** Image reconstruction settings.

Source	Series	Kernel	QIR Level	Slice Thickness, mm	Slice Increment, mm
NECT	TNC	Qr36	off	3.0	1.5
CTA	VNC^1^	Qr36	Q4	0.4	0.2
	VNC^2^	Br36	Q4	0.4	0.2
	VNC^3^	Qr36	Q4	1.0	0.4
	VNC^4^	Qr36	Q3	1.0	0.4

CTA = computed tomography angiography; NECT = non-enhanced computed tomography; QIR = quantum iterative reconstruction; TNC = true non-contrast; VNC = virtual non-contrast (including conventional and pure calcium).

**Table 2:** Baseline study characteristics.

Age, years	80.0 (75.3–82.8)
Sex, male	22/38 (57.9%)
BMI, kg/m^2^	27.7 ± 5.6
Cardiovascular risk factors:	
Arterial hypertension;	27/38 (71.1%)
Current or former smoker;	4/38 (10.5%)
Diabetes type 2;	17/38 (44.7%)
Hypercholesterolemia;	16/38 (42.1%)
Positive family history for adverse cardiovascular events;	1/38 (2.6%)
Obesity (BMI > 25 kg/m^2^).	10/38 (26.3%)
Coronary artery calcium:	
Total TNC Agatston score;	934 (167–1991)
Total TNC volume score, mm^3^.	811 (200–1623)

Normally distributed data shown as mean ± standard deviation; non-normally distributed data shown as median (interquartile range). BMI = body mass index.

## Data Availability

The data presented in this study are available on request from the corresponding author.
